# Integrated omics identifies VviGTs and VviMYB174 as drivers of glycosylated monoterpenoid accumulation in ‘Ruiduhongyu’ grapes

**DOI:** 10.1093/hr/uhag188

**Published:** 2026-05-08

**Authors:** Si-Yu Liu, Xiao-Miao Zhou, Song-Yu Liu, Xiao Meng, Ya-Chen Wang, Lei Sun, Chang-Qing Duan, Bao-Qing Zhu, Qiu-Hong Pan

**Affiliations:** Center for Viticulture and Enology, College of Food Science and Nutritional Engineering, China Agricultural University, Beijing 100083, China; Key Laboratory of Viticulture and Enology, Ministry of Agricultural and Rural Affairs, Beijing 100083, China; College of Biological Science and Biotechnology, Beijing Forestry University, Beijing 100085, China; College of Biological Science and Biotechnology, Beijing Forestry University, Beijing 100085, China; Center for Viticulture and Enology, College of Food Science and Nutritional Engineering, China Agricultural University, Beijing 100083, China; Key Laboratory of Viticulture and Enology, Ministry of Agricultural and Rural Affairs, Beijing 100083, China; Center for Viticulture and Enology, College of Food Science and Nutritional Engineering, China Agricultural University, Beijing 100083, China; Key Laboratory of Viticulture and Enology, Ministry of Agricultural and Rural Affairs, Beijing 100083, China; Institute of Forestry and Pomology, Beijing Academy of Agriculture and Forestry Sciences, Beijing 100093, China; Center for Viticulture and Enology, College of Food Science and Nutritional Engineering, China Agricultural University, Beijing 100083, China; Key Laboratory of Viticulture and Enology, Ministry of Agricultural and Rural Affairs, Beijing 100083, China; College of Biological Science and Biotechnology, Beijing Forestry University, Beijing 100085, China; Center for Viticulture and Enology, College of Food Science and Nutritional Engineering, China Agricultural University, Beijing 100083, China; Key Laboratory of Viticulture and Enology, Ministry of Agricultural and Rural Affairs, Beijing 100083, China

## Abstract

Monoterpenoids contribute to grape and wine varietal aroma as free volatiles and glycosidically bound precursors. While glycosylated forms largely determine postharvest aroma potential, the genes controlling their accumulation remain largely unidentified. Here, we compared free and glycosylated monoterpenoids and the accompanying transcriptome between the green-skinned ‘Ruiduxiangyu’ (RDXY) and the pink-skinned ‘Ruiduhongyu’ (RDHY), two siblings derived from the same cross. RDHY berries accumulated higher levels of glycosylated monoterpenoids during ripening, whereas RDXY berries were enriched in the free forms. Integrated metabolome–transcriptome analysis revealed a tight correlation between glycosylated monoterpenoid content and the expression of five glycosyltransferase (*GT*) genes (*VviGTs*) and the transcription factor *VviMYB174*. Besides the previously characterized VviGT14 and VviGT7, enzyme activity assay and transient overexpression experiments in tobacco leaves, grape leaves, and grape berries demonstrated that VviGT22, VviGT37, and VviGT74 also possess monoterpenol glycosylation activity. Yeast one-hybrid assays, combined with overexpression and silencing analyses, confirmed that VviMYB174 directly upregulates these three *GT* genes in RDHY, thereby promoting glycosylated monoterpenoid accumulation. Sequence alignment revealed that only *VviGT74* exhibited different promoter sequence divergence between the two cultivars, notably, the *VviGT74* promoter from RDXY failed to bind *VviMYB174*, which correlated with its reduced expression in RDXY berries. These findings provide new targets for manipulating the glycosylated aroma precursor pool and advance the molecular understanding of monoterpenoid metabolism in grapevine.

## Introduction

Grapevine (*Vitis vinifera* L.) is a major fruit crop extensively cultivated in China. Berry aroma is a critical determinant of grape quality and varietal character, profoundly influencing consumer acceptance. To date, over a 100 volatile compounds have been identified in grape berries, encompassing terpenoids, C13-norisoprenoids, esters, alcohols, and ketones [1, 2]. Among these, monoterpenoids are of particular significance due to their low olfactory thresholds and pleasant floral and citrus notes, which constitute the cornerstone of varietal aroma [3, 4]. Specific monoterpenols, such as linalool, citronellol, and geraniol, are recognized as primary contributors to the characteristic ‘Muscat’ flavor in grapes [5]. During berry development, monoterpenoids accumulate in both free and glycosidically bound forms [6, 7]. Free monoterpenoids are volatile and directly impart aroma, whereas glycosidically bound monoterpenoids are nonvolatile precursors that can be hydrolyzed to release volatile aglycones during winemaking through enzymatic or acid-catalyzed reactions, thereby enhancing wine fragrance complexity and intensity [3]. Consequently, the balance between free and glycosidically bound monoterpenoids not only shapes the aroma profile of grape berries but also determines the aromatic potential of the resulting wine [8]. Elucidating the biosynthetic mechanisms underlying glycosylated monoterpenoid formation in grapes is therefore essential for precision aroma enhancement in both grapes and wines.

Monoterpenoid biosynthesis in grape berries predominantly occurs through the plastid-localized methylerythritol phosphate (MEP) pathway. This pathway generates the universal C5 precursors isopentenyl diphosphate and dimethylallyl diphosphate, which are condensed by geranyl diphosphate synthase (GPPS) to form geranyl diphosphate (GPP). Subsequent conversion of GPP into diverse monoterpenoid compounds is catalyzed by terpene synthases (TPSs) [9]. In addition to the MEP pathway, NUDX (nucleotide diphosphate bound to X) hydrolases have been implicated in monoterpenoid metabolism in grapes [10]. The expression of several MEP pathway genes correlates with monoterpenoid accumulation, including 1-deoxy-D-xylulose-5-phosphate synthase (*VviDXS1* and *VviDXS3*) [11, 12] and geranylgeranyl pyrophosphate synthase large subunit (*VviGGPPS-LSU)*, the latter having been functionally validated to catalyze monoterpenoid synthesis *in vivo* [13]. To date, multiple TPS-b/g subfamily members, including VviTPS56 and VvTPS59, have been identified as functional monoterpenoid synthases [14–16]. Moreover, NUDX hydrolase VviNUDX1 and VviNUDX3 may participate in the monoterpenoid metabolism [17].

Following biosynthesis, free monoterpenoids are glycosylated by glycosyltransferases (GTs) to form nonvolatile glycosidic conjugates [18]. Members of the GT1 family are commonly designated as uridine diphosphate glycosyltransferases (UGTs) due to their utilization of UDP-glucose as the sugar donor. These enzymes harbor a conserved plant secondary product glycosyltransferase (PSPG) motif in their N-terminal region [19]. Several grape UGTs have been functionally validated to glycosylate monoterpenols *in vitro*, including VviGT7, VviGT14, VviGT15, and VviGT16, which catalyze the glycosylation of geraniol, citronellol, and nerol [20, 21]. Notably, VviGT7 exhibits sequence polymorphisms across grape cultivars, with critical amino acid substitutions leading to enzymatic inactivation [20]. Our previous work demonstrated that *VviGT7* and *VviGT14* expression positively correlates with monoterpenoid accumulation in ‘Gewürztraminer’ and ‘Muscat blanc à Petit grain’ berries [7]. Genome-wide analysis has classified VviUGTs into 15 distinct subfamilies [22]. Among them, the G subfamily, particularly VviUGT85A members, is frequently involved in monoterpenoid glycosylation. The CsGT1 homologs VviUGT85A30 and VviUGT85A33 have been shown to catalyze geraniol glycosylation *in vitro* [23]. Additionally, four other VviUGT85A members identified in grape leaves exhibit glycosylation activity toward linalool, geraniol, and α-terpineol [24]. Despite these advances, the contribution of UGTs from other subfamilies to glycosylated monoterpenoid formation remains largely unexplored.

At the transcriptional level, MYB transcription factors (TFs) play pivotal roles in regulating monoterpenoid biosynthesis across plant species. OfMYB21 promotes linalool synthesis by activating *OfTPS2* expression in *Osmanthus fragrans* flowers [25]. CsMYBs enhance linalool accumulation in tea leaves through regulation of *CsTPS76* [26]. Similarly, LcMYB44 upregulates *LcADH* to facilitate citral biosynthesis in *Litsea cubeba* [27]. In grapes, DAP-seq analysis revealed that VviMYB24 binds the promoter of *VviTPS35* and promotes monoterpenol accumulation [28]. Other TFs, including VvibZIP61 and VvibHLH9, have also been implicated in monoterpenoid regulation [29, 30]. Our previous studies demonstrated that VviERF003 and VviWRKY40 act as positive and negative regulators, respectively, of glycosylated monoterpenoid accumulation in berries through targeted upregulation of *VviGT14* expression [31, 32]. However, relative to the chemical diversity of monoterpenes and the size of the GT1 family, our understanding of glycosylated monoterpenoid accumulation in grape berries remains fragmentary.

The green-skinned ‘Ruiduxiangyu’ (RDXY) and the pink-skinned ‘Ruiduhongyu’ (RDHY) are two sibling cultivars derived from the same parental cross (*V. vinifera* Jingxiu × *V. vinifera* Xiangfei). ‘Jingxiu’, a hybrid cultivar developed in 1994 by the Beijing Botanical Garden, Chinese Academy of Sciences, from a cross of Hongzilu (female parent) and Pannonian (male parent), produces berries that ripen to a rosy-red or bright purplish-red color. By contrast, ‘Xiangfei’, a green-skinned cultivar with an intense Muscat aroma, was bred in 1982 by the Institute of Forestry and Pomology, Beijing Academy of Agriculture and Forestry Sciences, using a progeny of Muscat Hamburg and Pearl of Csaba as the female parent and Crimson as the male parent. Ripe RDXY berries exhibit a characteristic Muscat-flavor, whereas RDHY berries do not [33], a difference that may be attributed to variations in free monoterpenoid concentrations.

The present study investigated the accumulation patterns of free and glycosylated monoterpenoids, alongside transcriptome dynamics, during berry development in RDXY and RDHY. The objective was to identify key *VviGTs* and potential TFs governing glycosylated monoterpenoid accumulation through the integrated omics analysis. This work provides novel insights into the regulatory network of monoterpenoid metabolism in grapes.

## Results

### Divergent accumulation of free and glycosylated monoterpenoids between the two cultivars

**Figure 1:**
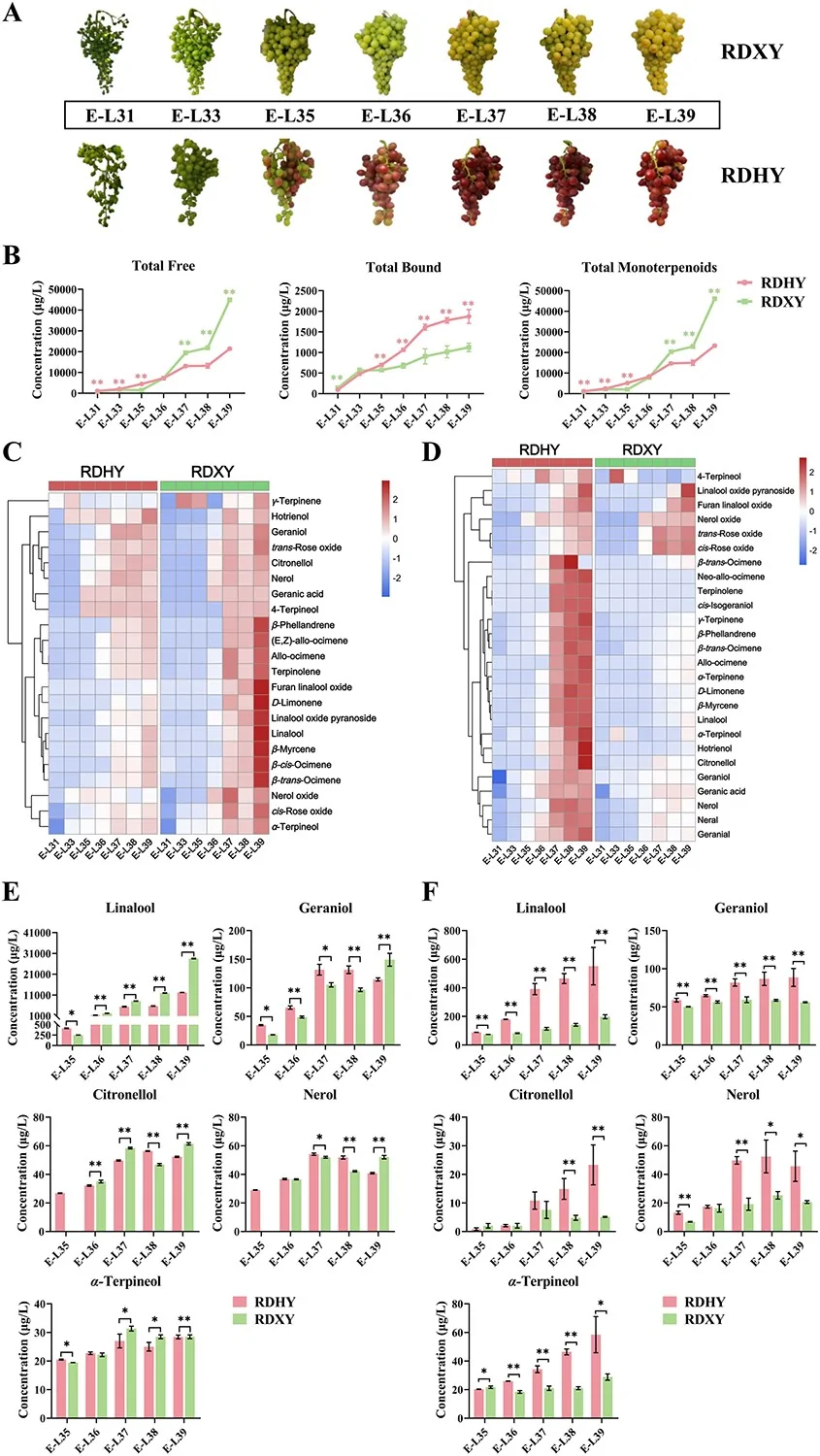
Dynamics of monoterpenoid accumulation during berry development in two grape cultivars. Samples from different development stages of ‘Ruiduhongyu’ (RDHY) and ‘Ruiduxiangyu’ (RDXY) (A). Concentrations of free, glycosylated-bound, and total monoterpenoids in RDHY and ‘Ruiduxiangyu’ RDXY. (B). Heatmaps showing individual monoterpenoid contents in free (C) and bound (D) forms. Concentrations of key differentially accumulated monoterpenes in free (E) and bound (F) pools. Data represent mean ± SD (*n* = 3). Asterisks indicate significant differences, ^*^, *P* < 0.05, **, *P* < 0.01.

We profiled monoterpenoid accumulation in RDXY and RDHY berries across seven developmental stages (E-L31, 33, 35–39) (Fig. 1A). In total, 22 free and 26 glycosylated monoterpenoids were identified (Supplementary Table S1). Both free and glycosylated monoterpenoids accumulated continuously during berry development, peaking at the E-L39 stage. As shown in Fig. 1B, free monoterpenoid content was significantly higher in RDXY than in RDHY from véraison onward, whereas glycosylated monoterpenoids showed the opposite trend, with greater abundance in RDHY during ripening. The total monoterpenoid profile largely mirrored that of the free forms, with overall higher levels in RDXY. Heatmap analysis revealed most free monoterpenoids were enriched in RDXY, particularly at E-L39, while the majority of glycosylated compounds accumulated to higher levels in RDHY during late development (Fig. 1C and D).

Principal component analysis demonstrated that the divergence in monoterpenoid profiles between the two cultivars emerged primarily from the véraison throughout full-ripening (E-L35–E-L39) (Supplementary Fig. S1). Accordingly, we investigated the dynamics of key ‘Muscat’ flavor-associated monoterpenoids during these five stages. Notably, free linalool, geraniol, nerol, citronellol, and α-terpineol were transiently more abundant in RDHY at véraison, but their subsequent accumulation accelerated in RDXY, resulting in significantly higher levels in RDXY by E-L39 (Fig. 1E). Conversely, the glycosylated forms of these compounds began diverging markedly from E-L37, with the disparity intensifying as maturation progressed (Fig. 1F). This differential accumulation of glycosylated monoterpenoids likely contributes to the distinct ‘Muscat’ aroma intensity between RDHY and RDXY.

### Genome-wide identification and developmental expression profiling of *VviUGTs*

To identify genes underlying the differential accumulation of glycosylated monoterpenoids, RNA-seq was performed on berries of RDXY and RDHY across seven developmental stages. A total of 42 cDNA libraries were constructed; sequencing statistics are provided in Supplementary Table S2. After quality control, 291.05 Gb of clean reads were retained, with Q30 ≥ 89.08% for every library. Over 85% of reads mapped to the grape reference genome (PN40024), yielding 43 453 annotated genes.

**Figure 2:**
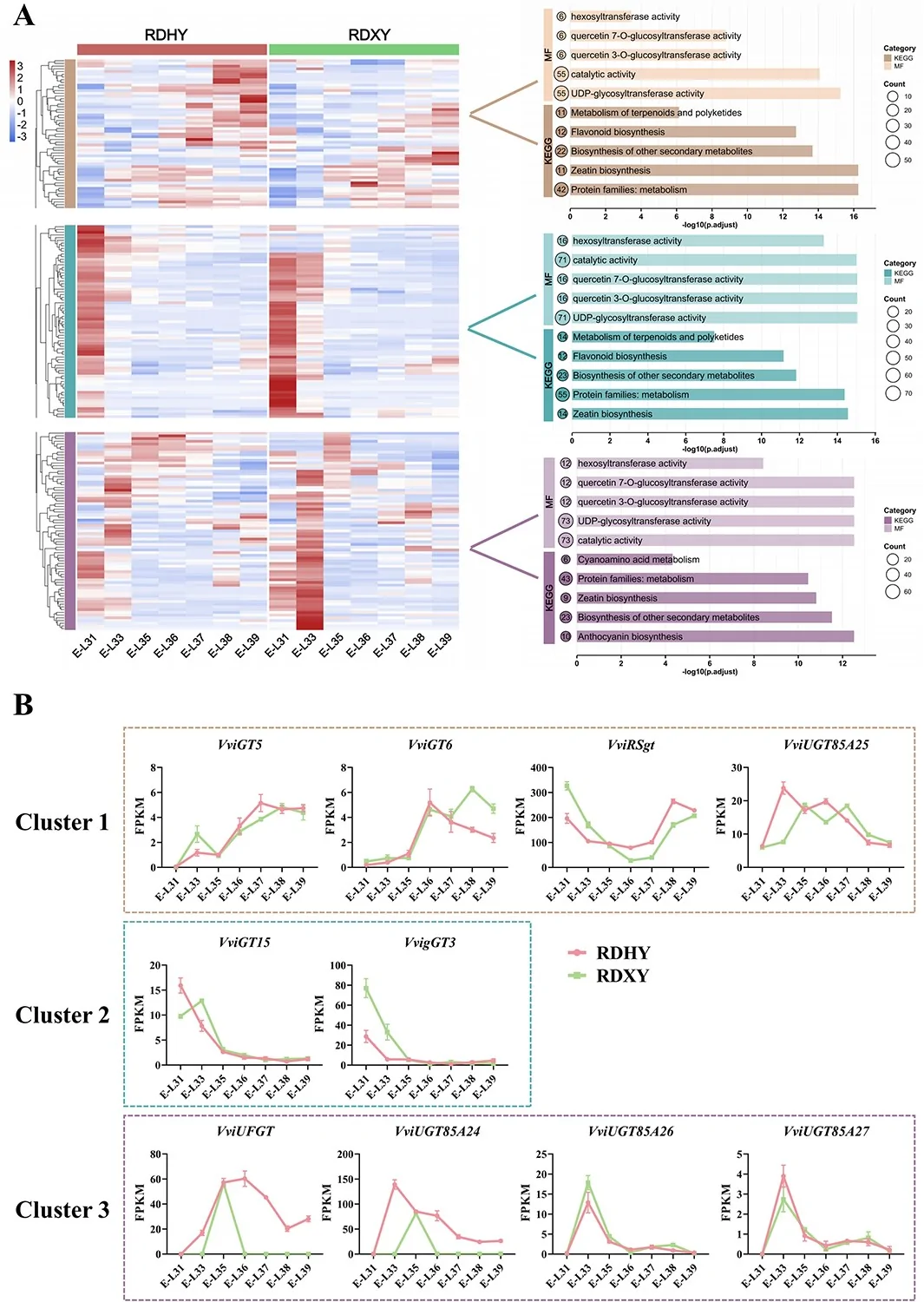
The expression pattern of *VviUGTs* in ‘Ruiduhongyu’ (RDHY) and ‘Ruiduxiangyu’ (RDXY). Clustering analysis of *VviUGTs* expression levels, functional enrichment (GO and KEGG) of *VviUGTs* (A). Expression profile of functionally characterized *VviUGTs* in different clusters (B).

Combining functional annotation with literature curation identified 233 putative *VviUGTs*, of which 199 were expressed in at least one of the two cultivars (Supplementary Table S3). Hierarchical clustering of these 199 UGTs resolved three distinct temporal patterns (Fig. 2A).

Cluster 1 (55 genes) showed elevated expression during late ripening (E-L 37–39) in both cultivars. Functional enrichment indicated significant association with flavonoid and terpenoid metabolism, and included *VviGT5* and *VviGT6* (flavonol glycosylation) [34], *VviRSgt* (resveratrol and hydroxycinnamic acid glycosylation) [35], and *VviUGT85A25* (monoterpenol glycosylation) [24] (Fig. 2B). Notably, *VviUGT85A25* peaked at prevéraison or véraison, a profile that does not mirror the late accumulation of glycosylated monoterpenoids.

Cluster 2 (71 genes) was maximally expressed at early developmental stage (E-L 31), and declined thereafter; *VvigGT3* (phenolic acid glycosylation) [36] and *VviGT15* (monoterpene glycosylation) [21] were representative members (Fig. 2B).

Cluster 3 (73 genes) displayed a sharp upregulation at E-L33, especially in RDXY. KEGG enrichment pointed to anthocyanin biosynthesis, consistent with the presence of *UFGT*, the final step enzyme in anthocyanin formation (Fig. 2B). Three additional *VviUGT85A* genes were grouped here; they were highly expressed at prevéraison or véraison but nearly silent during ripening, implying a minimal role in berry glycosylated monoterpenoid accumulation.

### Candidate gene screening for monoterpenoid glycosyltransferases

Because the cultivar-specific difference in monoterpenoid content became pronounced from véraison onward, we restricted the search for regulatory genes to the five stages E-L35–39. With the thresholds of |log_2_FoldChange| ≥ 1 and FDR < 0.01, a total of 3481 differentially expressed genes (DEGs) were detected; the largest sets occurred at E-L38 and E-L39 (Supplementary Fig. S2). To determine whether upstream biosynthetic steps drive the divergence, we first inspected the MEP and mevalonic acid (MVA) pathways. Among the 55 annotated genes (including the functionally characterized *VviIDI* [37] and *VviGGPPS_LSU* [13]), none were differentially expressed between cultivars (Supplementary Table S4). Likewise, of 36 TPS-b/g and 25 NUDX members, only *VviTPS35*, *VviNUDX18*, and *VviNUDX21* were differentially expressed, all with higher transcript levels in RDHY—a trend inverse to that of free monoterpenoid accumulation. We therefore postulated that differential glycosylation, rather than *de novo* synthesis, underlies the observed pattern.

**Figure 3:**
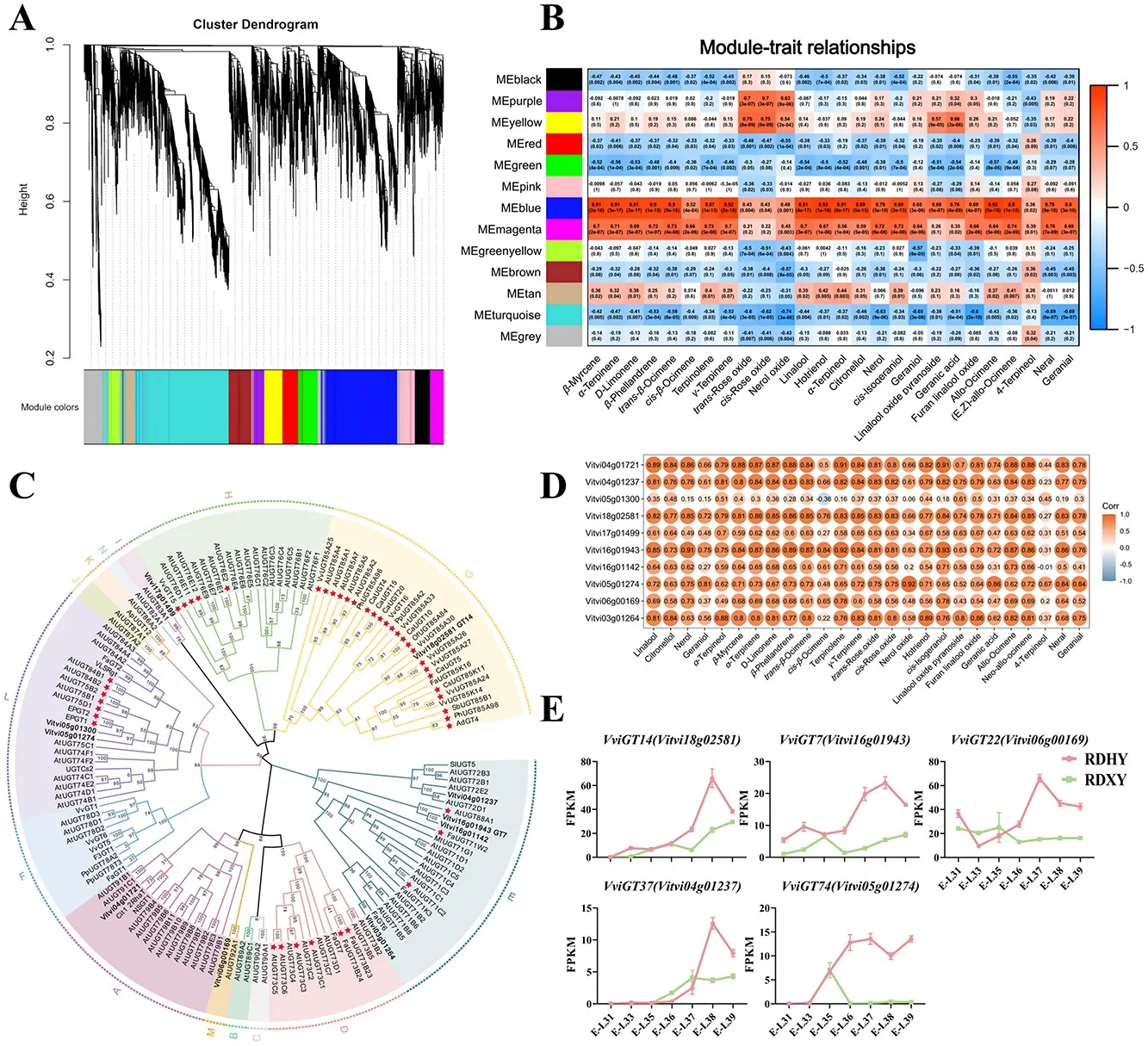
WGCNA of DEGs and candidate UGTs mining. (A) Hierarchical cluster tree revealing coexpression modules identified. (B) Correlation between module eigengenes and content of bound-form monoterpenoids. (C) Phylogenetic tree of candidate UGTs with UGTs from *A. thaliana* and other species, the star represents monoterpene UGTs. The NCBI accession numbers are provided in Supplementary Table S6. (D) Correlation between the expression of candidate UGTs and the abundance of glycosidically bound monoterpenoids in RDHY berries. (E) The expression levels of five candidate UGTs in both cultivars.

To identify GTs potentially responsible for this conversion, weighted gene coexpression network analysis (WGCNA) was performed using the 3481 DEGs and the glycosylated monoterpenoid content of both cultivars. Thirteen coexpression modules were resolved (Fig. 3A). The MEblue and MEmagenta modules displayed strong positive correlations (correlation coefficient > 0.6) with majority of glycosylated monoterpenoids (Fig. 3B), so they were mined for UGTs. Ten candidates were retained (Supplementary Table S5), including the previously characterized *VviGT7* and *VviGT14*. All possess the conserved PSPG motif (Supplementary Fig. S3).

Phylogenetic analysis placed *Vitvi04g01237*, *Vitvi05g01274*, *Vitvi03g01264*, *Vitvi16g01142*, and *Vitvi05g01300* in clades containing functionally proven monoterpenoid UGTs from other species (Fig. 3C). Expression patterns of nine candidates (all except *Vitvi05g01300*) positively correlated with glycosylated monoterpenoid accumulation in RDHY (Fig. 3D). Filtering for fragments per kilobase of transcript per million mapped reads (FPKM) > 5 (Supplementary Fig. S4) yielded five high-confidence genes—*VviGT14*, *VviGT7*, *Vitvi06g00169*, *Vitvi04g01237*, and *Vitvi05g01274*—each strongly upregulated during late ripening in RDHY (Fig. 3E).

### Functional characterization of candidate VviUGTs

**Figure 4:**
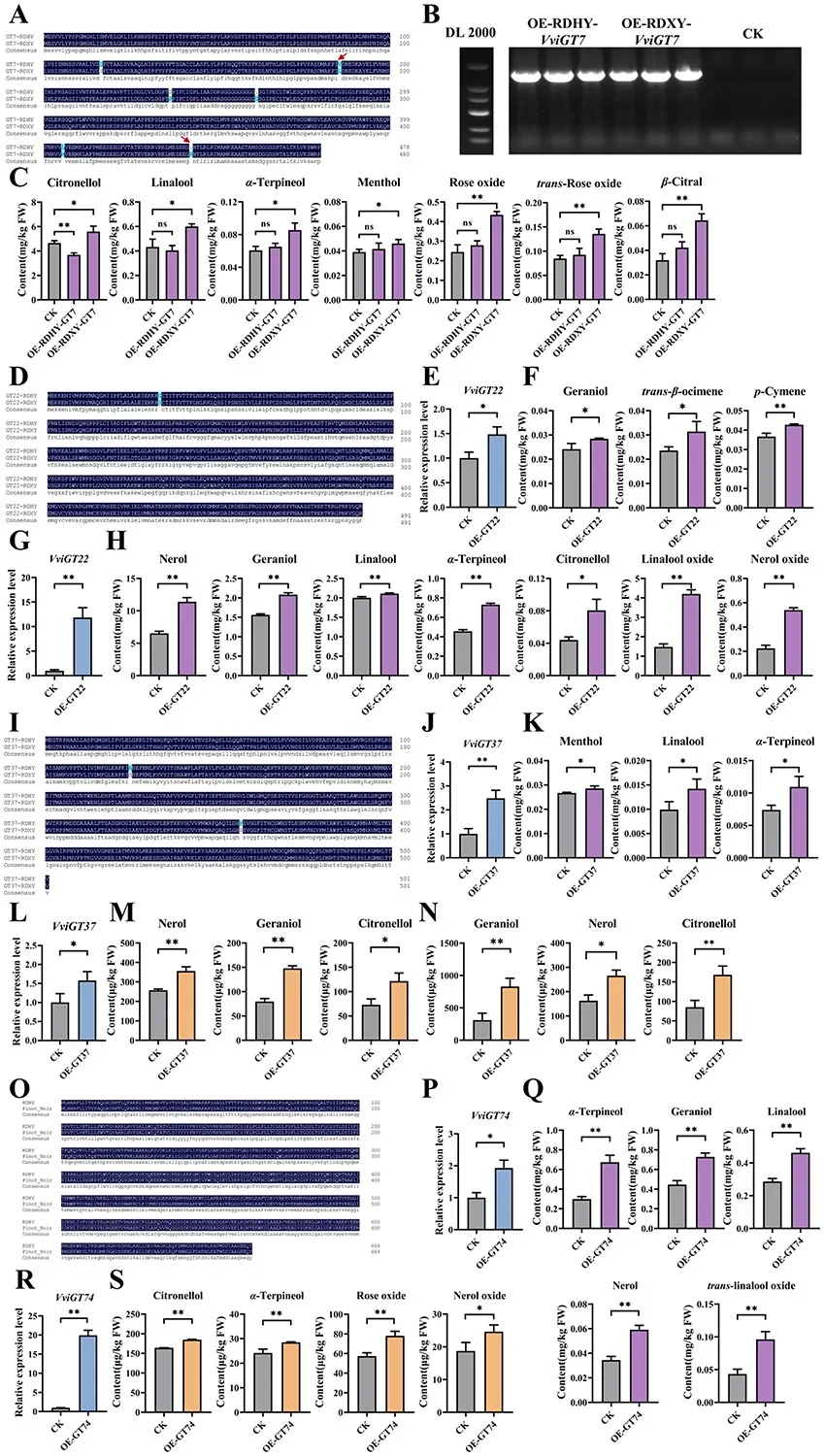
Sequence alignment and functional characterization of candidate UGTs. The protein sequence comparison of VviGT7 (A), VviGT22 (D), and VviGT37 (I) between two cultivars. The protein sequence comparison of VviGT74 between RDHY and Pinot Noir (PN40024 12X.v3) (O). The expression of *VviGT7* in tobacco leaves (B). The content of glycosylated monoterpenoids in *OE-VviGT7* tobacco leaves (C). The expression levels of *VviGT22* in OE *V. vinifera* Marselan (E) and *V. quinquangularis* (G) leaves. The content of glycosylated monoterpenoids in *OE-VviGT22* ‘Marselan’ (F) and *V. quinquangularis* leaves (H). The expression levels of *VviGT37* in OE ‘Marselan’ leaves (J) and ‘Summer Black’ grape skins (L). The content of glycosylated monoterpenoids in *OE-VviGT37* ‘Marselan’ leaves (K) and ‘Summer Black’ grape skins (M). The content of free-form monoterpenoids in *OE-VviGT37* ‘Summer Black’ grape skins (N). The expression levels of *VviGT74* in OE *V. vinifera* Ruidukemei leaves (P) and ‘Sable’ grape skins (R). The content of glycosylated monoterpenoids in *OE-VviGT74* ‘Ruidukemei’ leaves (Q) and ‘Sable’ grape skins (S).

To determine whether coding-sequence divergence contributes to the cultivar-specific glycosylation phenotype, all five candidate UGTs were cloned from RDHY and RDXY. We initially examined *VviGT14* and *VviGT7*, both previously reported to glycosylate monoterpenol *in vitro*. The *VviGT14* open reading frame was identical between cultivars. Transient overexpression in RDHY berries significantly elevated glycosylated geraniol, nerol, linalool, and citronellol, and also increased free linalool and α-terpineol (Supplementary Fig. S5, Supplementary Table S7). By contrast, the *VviGT7* allele differed between cultivars at two positions: Leu → 186 → Val in RDHY and a single-Arg insertion after position 443 in RDXY (Fig. 4A). To assess the impact of these substitutions, both alleles were expressed in tobacco leaves supplied with a monoterpenol mixture (Fig. 4B). Gas chromatography-mass spectrometry (GC–MS) detected 16 glycosylated products (Supplementary Table S8); almost all accumulated highly in tobacco leaves expressing the RDXY allele, whereas no significant increase was observed in leaves expressing the RDHY allele compared with the control (Fig. 4C). Thus, the L186V mutation abolishes VviGT7 *in vivo* activity, indicating that this isoform does not drive the higher glycosylated monoterpenoid content observed in RDHY.

Three previously uncharacterized UGTs were subsequently analyzed. *VviGT22* (*Vitvi06g00169*) showed 99.8% nucleotide identity between cultivars (Fig. 4D). Transient overexpression in *V. vinifera* Marselan leaves significantly raised glycosylated geraniol and also increased free linalool, citronellol, and geraniol (Fig. 4E and F, Supplementary Table S9). Substrate-feeding assays in *Vitis quinquangularis* and tobacco leaves confirmed elevated levels of all detectable glycosylated monoterpenoids (Fig. 4G and H, Supplementary Table S10, and Supplementary Fig. S6).

*VviGT37* (*Vitvi04g01237*) was likewise highly conserved (Fig. 4I). Transient overexpression in ‘Marselan’ leaves increased glycosylated menthol, linalool, and α-terpineol, and augmented free geraniol and citronellol (Fig. 4J and K, Supplementary Fig. S7). Validation in ‘Summer Black’ grape skins showed elevated free and glycosylated nerol, geraniol, and citronellol (Fig. 4L–N), confirming monoterpenol glycosylation activity in fruit tissue.

*VviGT74* (*Vitvi05g01274*) was cloned only from RDHY, its sequence matched the reference genome exactly (Fig. 4O). Overexpressing in *V. vinifera* Ruidukemei leaves significantly raised both free and glycosylated α-terpineol, linalool, and geraniol (Fig. 4P and Q). Tobacco assays corroborated these results (Supplementary Fig. S8), and expression in ‘Sabel’ grape skins increased glycosylated citronellol, α-terpineol, rose oxide, and nerol oxide (Fig. 4R and S).

To assess the *in vitro* enzymatic function of these VviGTs, we performed activity assays using the UDP-Glo™ Glycosyltransferase system. This assay quantifies GT activity by measuring UDP release upon glycoside bond formation. Compared with both the negative control (reaction without enzyme) and the MBP tag-only control, UDP levels were significantly elevated in reaction systems containing VviGT22, VviGT37, or VviGT74 (using UDP-glucose and linalool as substrates), demonstrating that three enzymes possess monoterpenol GT activity *in vitro* (Supplementary Fig. S9).

Collectively, the data demonstrate that VviGT22, VviGT37, and VviGT74 are efficient monoterpenol GTs and represent key contributors to the formation of glycosylated monoterpenoid aroma precursors in the studied grape berries.

### VviMYB174 positively regulates glycosylated monoterpenoid accumulation

**Figure 5:**
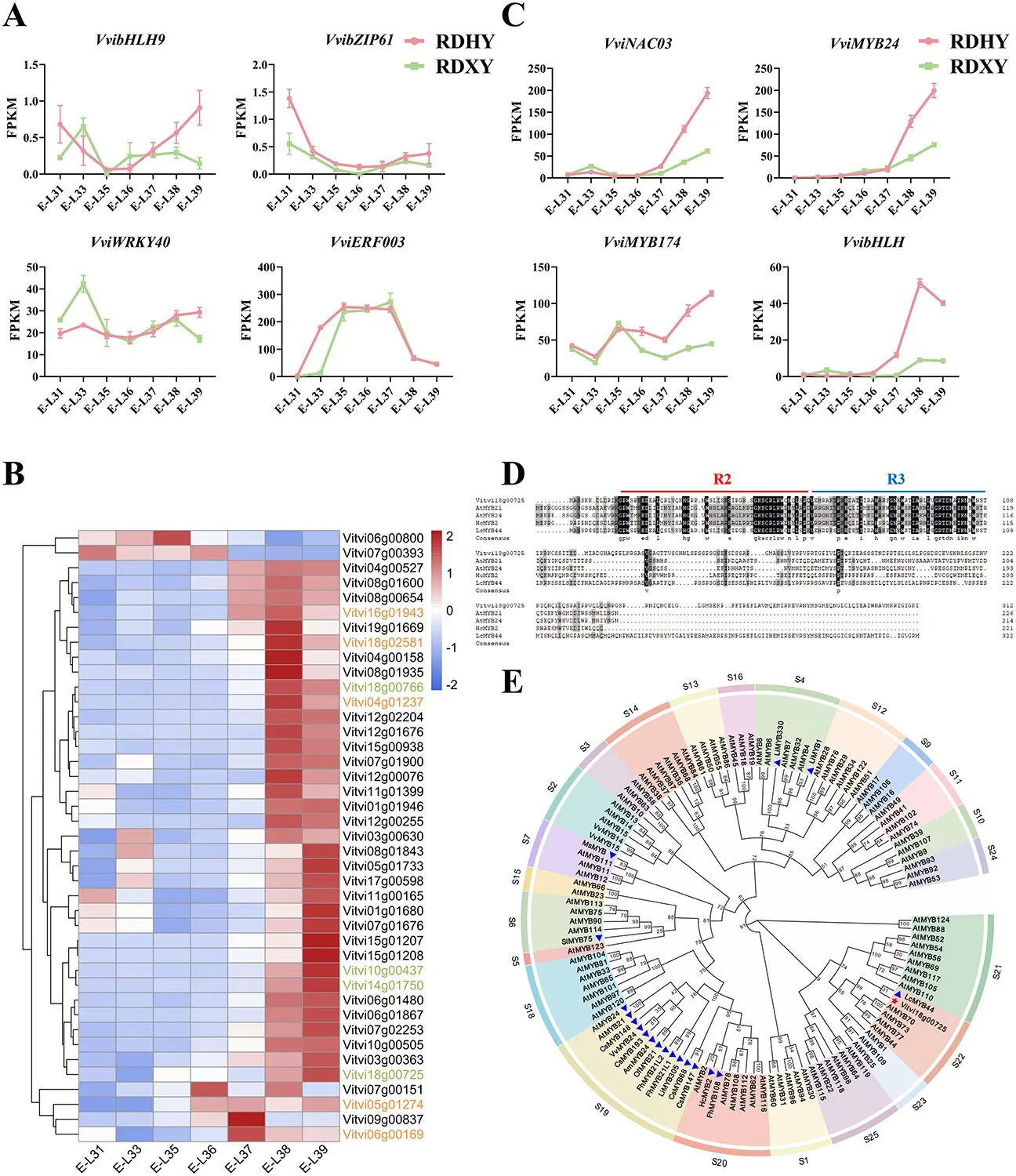
Screening of candidate TFs that potentially regulate glycosylated monoterpene biosynthesis. (A) The expression pattern of TFs that has been reported to modulate monoterpene accumulation. (B) Clustering heatmap of candidate *TFs* and *UGTs* expression levels in RDHY. (C) The expression profile of four key candidate TFs in two cultivars. (D) Multiple sequence alignment of VviMYB174 and other R2R3-MYBs. (E) Phylogenetic tree of VviMYB174 with R2R3-MYBs in *A. thaliana* and other species. The triangle denotes TFs that can regulate terpenoid synthesis, as detailed in Supplementary Table S13.

The biosynthesis of monoterpenoids in plants is often transcriptionally regulated. We first examined the expression of TFs previously implicated in grape monoterpenoid metabolism. Among these, *VvibHLH9* and *VvibZIP61* were expressed at low levels in both cultivars and were primarily active during early developmental stages (Fig. 5A). Although *VviERF003* (reported to activate glycosylated monoterpenoid accumulation) and *VviWRKY40* (a repressor) were expressed at relatively high levels, their transcript abundance peaked around véraison, a pattern that does not align with the observed accumulation of glycosylated monoterpenoids in our study (Fig. 5A). Moreover, none of these four TFs showed cultivar-specific expression, indicating that they do not underlie the phenotypic difference we observed.

We therefore employed WGCNA to identify candidate regulatory TFs. From the MEblue and MEmagenta modules, we retrieved 36 TFs (Supplementary Table S5). MYB family members were most abundant (13 members), followed by bHLH (5 members) and NAC (4 members). We then clustered the expression profiles of these 36 TFs together with the five candidate UGTs and retained TFs whose patterns tracked those of the UGTs (Fig. 5B). Applying an expression threshold (FPKM > 50) filtered this set to four high-confidence candidates: *Vitvi10g00437* (*VviNAC03*), *Vitvi14g01750* (*VviMYB24*), *Vitvi18g00725* (*VviMYB174*), and *Vitvi18g00766* (*VvibHLH*). Among them, VviMYB24 has been previously reported to regulate monoterpene biosynthesis in grape berries. All four TFs were highly expressed during late berry development, consistent with the accumulation pattern of glycosylated monoterpenoids (Fig. 5C).

Given the central role of MYB TFs in monoterpenoid regulation, we focused on VviMYB174. Multiple sequence alignment confirmed the presence of the R2–R3 DNA-binding domain, characteristic of R2R3-MYB proteins (Fig. 5D). Phylogenetic analysis with *Arabidopsis* and other plant R2R3-MYBs placed VviMYB174 in the S22 subfamily, where it clusters with LcMYB44 from *L. cubeba*, an activator of citral biosynthesis (Fig. 5E).

**Figure 6:**
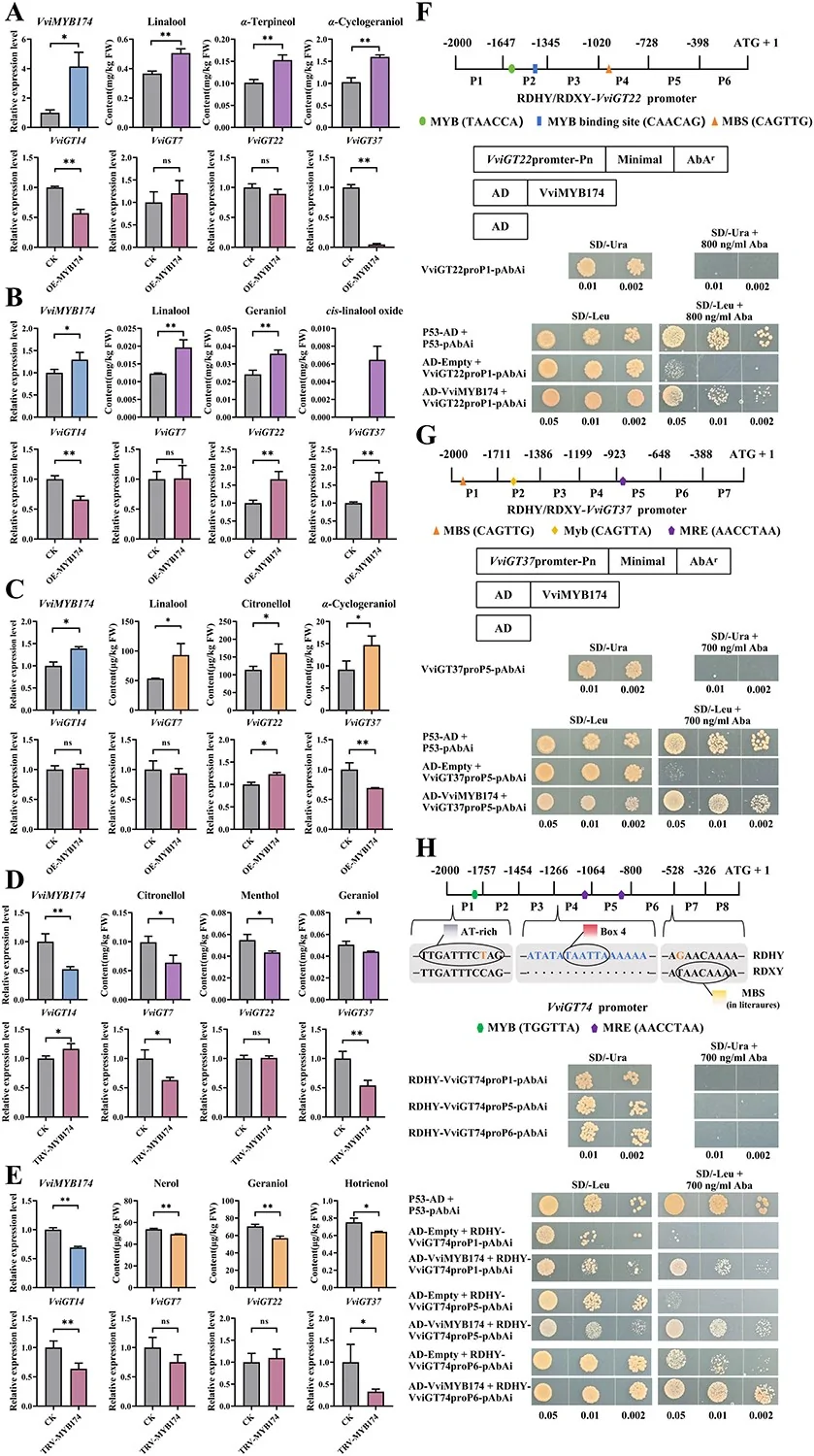
VviMYB174 promotes monoterpenoid biosynthesis by directly binding to the promoter of *VviGTs*. Expression levels of *VviMYB174* and related *VviGTs* and monoterpenoids accumulation in OE *V. vinifera* Ruidukemei leaves (A), *V. vinifera* Marselan leaves (B), and grape skins (C). Expression levels of *VviMYB174* and related *VviGTs* and monoterpenoids accumulation in VIGS *V. vinifera* Marselan leaves (D) and RDHY berries (E). Promoter cis-acting elements analysis and binding analysis of VviMYB174 to the promoter of *VviGT22* (F), *VviGT37* (G), and *VviGT74* (H).

To test VviMYB174 function, we performed transient overexpression and virus-induced gene silencing (VIGS) assays in multiple grape tissues and quantified the expression of the four consistently detectable *VviGTs*. Overexpression of *VviMYB174* in *V. vinifera* Ruidukemei leaves significantly elevated glycosylated linalool, α-terpineol, and α-cyclogeraniol, despite decreased expression of *VviGT14* and *VviGT37* (Fig. 6A, Supplementary Table S11). Comparable effects were observed in *V. vinifera* Marselan leaves, where overexpression boosted glycosylated geraniol and linalool, aligning with the elevated expression of *VviGT22* and *VviGT37* (Fig. 6B). Further validation in ‘Sabel’ grape skins showed that VviMYB174 promoted the accumulation of glycosylated linalool, citronellol, and α-cyclogeraniol, concomitant with a marked upregulation of *VviGT22* expression (Fig. 6C, Supplementary Table S12). Conversely, silencing *VviMYB174* in ‘Marselan’ leaves significantly reduced glycosylated citronellol, geraniol, and menthol (Fig. 6D). A parallel reduction in glycosylated nerol, geraniol, and hotrienol was observed in *VviMYB174*-silenced RDHY berries (Fig. 6E). Notably, *VviGT37* expression was significantly downregulated in both silenced systems (Fig. 6D and E). Beyond glycosidically bound forms, both overexpression and silencing of *VviMYB174* also perturbed free monoterpenoid pools, particularly monoterpenols (Supplementary Fig. S10). These findings establish VviMYB174 as a positive regulator of glycosylated monoterpenoid accumulation in grape berries.

To determine whether VviMYB174 directly regulates the four *GT* genes, we performed yeast one-hybrid (Y1H) assays. Promoter sequences of *VviGT14*, *VviGT22*, *VviGT37*, and *VviGT74* were cloned from both the RDHY and RDXY genomes and analyzed for cis-acting elements using PlantCARE, which identified potential MYB-binding sites in all four promoters (Fig. 6F–H, Supplementary Figs S11–S14). Sequence alignment revealed that apart from *VviGT74*, the promoters of the other three genes were identical between the two cultivars. In contrast, the *VviGT74* promoter exhibited extensive sequence variation, including insertions, deletions, and single-nucleotide polymorphisms. These variations resulted in the gain of an AT-rich element and a Box 4 element in the RDHY promoter, concomitant with the loss of a previously reported MBS motif (Fig. 6H, Supplementary Fig. S14).

Y1H analysis revealed distinct DNA-binding patterns of VviMYB174 to these promoters: no interaction was detected with the *VviGT14* promoter (Supplementary Fig. S15); VviMYB174 bound the P1 fragment of the *VviGT22* promoter, even though this region lacks a recognizable MYB core motif (Fig. 6F); binding to the *VviGT37* promoter was specific to the P5 fragment, which harbors an MRE motif (Fig. 6G); and VviMYB174 exhibited the most extensive binding to the *VviGT74* promoter from RDHY, interacting with the P1, P5, and P6 fragments, two of which containing predicted MYB-binding sites (Fig. 6H). In contrast, no detectable interactions were observed between VviMYB174 and the *VviGT74* promoter from RDXY, despite the presence of an MBS motif in the P6 fragment (Fig. 6H, Supplementary Fig. S16).

## Discussion

Monoterpenols impart the characteristic ‘Muscat’ note to grape berries, yet their ultimate sensory impact is dictated by the ratio of free (volatile) to glycosidically bound (nonvolatile) forms [18, 38]. We observed that RDHY accumulates up to 1.8-fold higher glycosylated monoterpenoids and 2.0-fold lower free monoterpenoids in comparison to RDXY. This metabolic phenotype correlates tightly with the concerted upregulation of four functional GTs—*VviGT14*, *VviGT22*, *VviGT37*, *VviGT74*—and the R2R3-MYB activator *VviMYB174* during late ripening.

### Cultivar-specific monoterpenoid signatures arise from differential *VviGT*s expression

The expression of the structural genes is relevant to the monoterpenoid accumulation in plants. Previous studies have established that rate-limiting enzymes in the terpenoid backbone pathway VviDXS1/3, isopentenyl pyrophosphate isomerase VviIDI*,* and geranylgeranyl pyrophosphate synthase large subunit VviGGPPS_LSU all contribute to monoterpenoid levels in grape berries [12, 13, 37]. As key enzymes in the final step of terpenoid biosynthesis, TPS-b/g members directly determine monoterpenoid composition [14]. However, our transcriptome data demonstrate that the differential concentration of monoterpenoids between cultivars is not attributable to the MEP pathway genes or majority of TPSs, except for *VviTPS35* (Supplementary Table S4). The elevated expression of this gene in RDHY, coupled with its lower free monoterpenoid levels, indicates rapid glycosylation of newly synthesized compounds. Monoterpenoid glycosylation is catalyzed by UDP-GTs. While several UGTs, including VviGT15, VviGT16, and some VviUGT85A members show monoterpenol glycosylation activity *in vitro*, their expression patterns did not correlate with glycosylated monoterpenoid accumulation in these cultivars (Fig. 2B, Supplementary Table S3). This indicates they are not primary drivers of the observed metabolic variation. We also evaluated VviGT14 and VviGT7, both previously linked to glycoside accumulation in berries [7, 39, 40]. Although *VviGT7* expression was higher in RDHY, a cultivar-specific L186V substitution impaired the activity *in vivo*, disqualifying it as a contributor to the RDHY phenotype (Fig. 4A–C). In contrast, *VviGT14* only showed differential expression between cultivars and could catalyze monoterpenol glycosylation in *planta* (Supplementary Fig. S5).

Monoterpenol glycosylation has been documented in *Arabidopsis* through UGTs from subfamilies D, E, G, L, and H [41]. Beyond the previously studied VviGT7 and VviGT14, we identified three additional candidates whose expression profiles mirror the accumulation of glycosylated monoterpenoids in RDHY. *In vitro* and *in vivo* functional assays confirmed that VviGT37 from subfamily E, VviGT74 from subfamily L, and VviGT22 from subfamily M all enable to elevate glycosylated monoterpenols (Fig. 4, Supplementary Fig. S9). Sequence-level data rule out neofunctionalization as the source of variation: the open reading frames of *VviGT14*, *VviGT22*, and *VviGT37* are almost identical between RDHY and RDXY, and allelic products display the same substrate spectrum *in vivo*. The decisive difference is transcriptional dosage. From véraison onward these three genes and *VviGT74* constitute a coexpression module whose aggregate abundance scales directly related to the cultivar-specific accumulation of glycosylated monoterpenoids. Thus, the observed cultivar contrast is governed by quantitative upregulation of a small UGT battery during ripening rather than by the acquisition of new catalytic functions.

### VviMYB174 orchestrates a metabolic shift toward glycosylated monoterpenoids

MYB-TFs play important roles in regulating the biosynthesis of anthocyanins, flavonoids, stilbenes, and terpenoids. R2R3-MYB subfamily members have been repeatedly implicated in monoterpenoid regulation across species—for instance, MsMYB represses the expression of *MsGPPS.LSU* in spearmint [42], while FhMYB21/FhMYB108 [43] and LiMYB108/LiMYB305 [44, 45] activate *TPS* in *Freesia hybrida* and *Lilium* ‘Siberia.’ HcMYB2 activates linalool biosynthesis in *Hedychium coronarium* flowers [46]. Homologs of MYB24 in snapdragon also contribute to monoterpenoid regulation [47], and in grapevine, VviMYB24 directly binds the *VviTPS35* promoter to enhance free monoterpenoid synthesis [28]. In this study, we found its expression correlated with glycosylated monoterpenoid abundance (Fig. 5B and C), suggesting it may also influence the glycosylation step, perhaps by modulating *VviGTs* expression.

VviMYB174, an R2R3-MYB most closely related to the monoterpenoid regulator LcMYB44 from *L. cubeba* [27], emerged from our network analysis as a strong candidate. Overexpression and virus-induced silencing in grape leaves and berries demonstrated that VviMYB174 positively regulates glycosylated monoterpenoid accumulation, yet the expression changes of downstream *VviGTs* were inconsistent (Fig. 6A–E). This complexity is consistent with its reported lack of intrinsic transactivation activity [48], implying a mechanism that may involve partnership with other TFs or coregulators—a hypothesis consistent with our observation that the TF does not work alone. Notably, VviMYB174 also negatively regulates anthocyanin biosynthesis by targeting *VviDFR* and *VviUFGT* and by competing with VviWDR2 for MBW complex assembly [48]. Our study also found that VviMYB174 binds to the promoters of *VviGT22*, *VviGT37*, and the promoter of *VviGT74* from RDHY (Fig. 6F–H). Further promoter sequence analysis revealed that the *VviGT74* promoter harbors substantial sequence variation between RDHY and RDXY, resulting in the loss of VviMYB174 binding to the RDXY allele (Supplementary Figs S14–S16). These findings support the notion that natural promoter variation may account for cultivar-specific gene expression [49]. However, whether VviMYB174 acts through interaction with VvibHLH9, VvibZIP61, VviERF003, and VviWRKY40 [30–33] remains to be determined.

**Figure 7:**
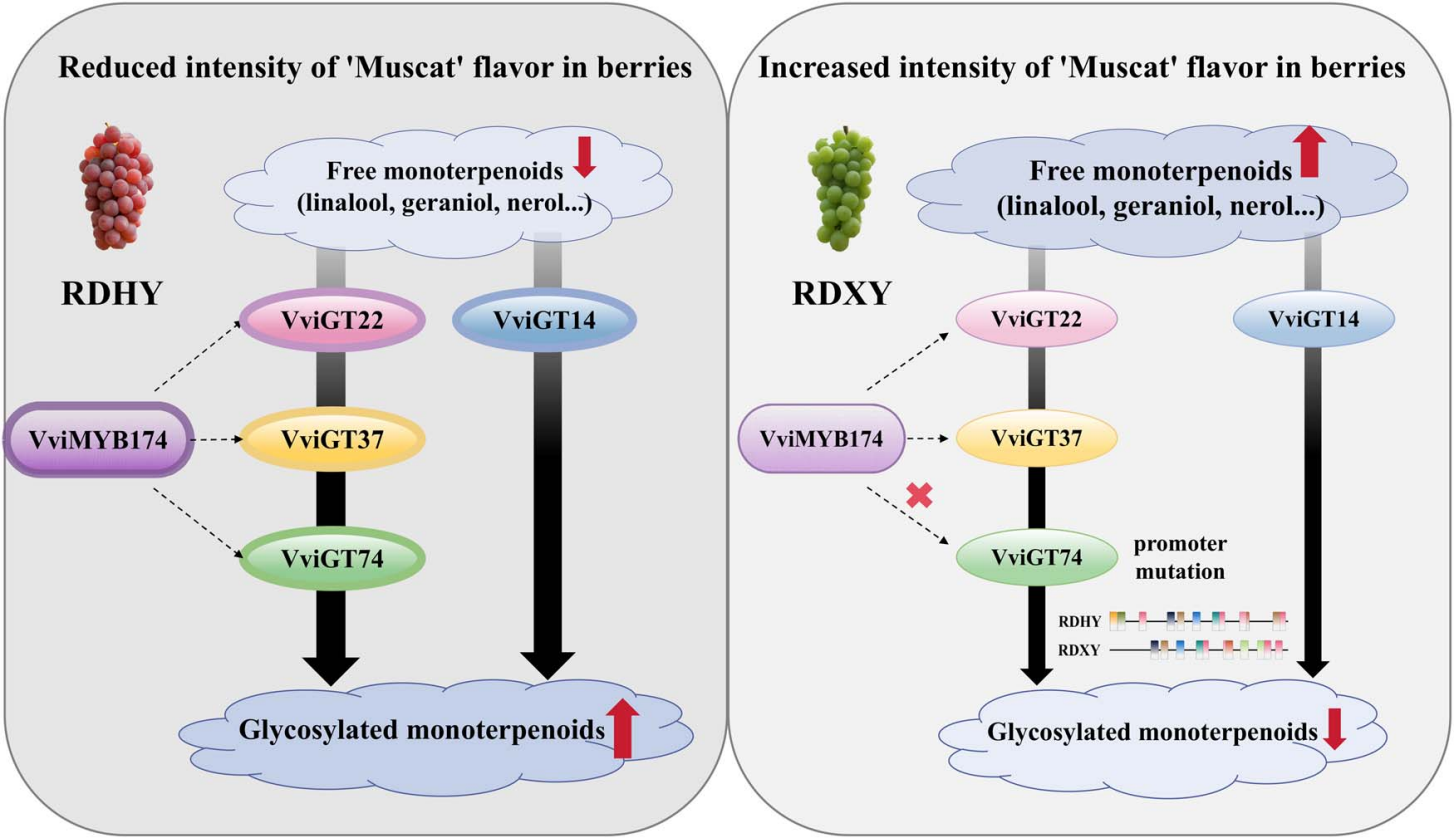
A model for the molecular basis underlying differential monoterpenoid glycosylation and ‘Muscat’ flavor intensity in RDHY and RDXY berries. In ripening RDHY berries, highly expressed VviMYB174 directly upregulates *VviGT22*, *VviGT37*, and *VviGT74*, and *VviGT14* is highly expressed independently. Together, these active GTs efficiently convert free monoterpenoids into glycosylated forms, leading to reduced free monoterpenoids and decreased ‘Muscat’ flavor intensity. In ripening RDXY berries, a promoter mutation in *VviGT74* (sequence variations shown in alignment) abolishes its binding with VviMYB174 (marked with ×), resulting in reduced expression of *VviGT74*. Additionally, *VviMYB174* shows reduced expression, leading to downregulation of *VviGT37* and *VviGT22*. Consequently, the overall glycosylation capacity is diminished, causing accumulation of free monoterpenoids and increased ‘Muscat’ flavor intensity.

In summary, our results elucidate the molecular mechanism underlying distinct monoterpenoid metabolism between RDXY and RDHY (Fig. 7). In ripening RDHY, *VviMYB174* was highly expressed*,* which promotes glycosylated monoterpenoid accumulation by directly upregulating *VviGT22*, *VviGT37*, and *VviGT74*. Together with highly expressed *VviGT14,* these four functional GTs drive efficient conversion of free monoterpenoids. In RDXY, *VviMYB174* exhibited reduced expression, and only upregulates *VviGT22* and *VviGT37* due to natural promoter mutations in *VviGT74*. Combined with the lower expression of *VviGT14*, less free monoterpenoids are converted into glycosylated forms. Consequently, RDHY berries accumulates more glycosylated monoterpenoids than RDXY, resulting in a less intense ‘Muscat’ flavor. These findings advance our understanding of monoterpenoid regulation and provide a genetic framework for tailoring aroma profiles in grapes.

## Materials and methods

### Plant materials

Berries of *V. vinifera* ‘Ruiduhongyu’ (RDHY) and ‘Ruiduxiangyu’ (RDXY) were collected in 2020 at the Institute of Forestry and Pomology, Beijing Academy of Agriculture and Forestry Sciences, located in Beijing, China (39 58 N, 116 13 E). Samples were harvested at seven developmental stages according to the E-L system: E-L31, 33, 35–39 [50]. For each cultivar and stage, 100 berries were collected, immediately frozen in liquid nitrogen, and stored at −80°C until analysis.

For transient transformation assays, RDHY berries at the E-L35 stage and leaves of *V. quinquangularis* L. were obtained from the Institute of Forestry and Pomology, Beijing Academy of Agriculture and Forestry Sciences in 2025. Leaves of *V. vinifera* Marselan and Ruidukemei, and berries of ‘Summer Black’ and ‘Sabel’ were collected from the Zhuozhou Experimental Station, China Agricultural University, in 2025. Tobacco (*Nicotiana benthamiana*) seedlings were grown for seven 7 weeks in a greenhouse under a 16-h light/8-h dark photoperiod at 25°C.

### Determination of free and glycosylated monoterpenoids

Free and glycosylated monoterpenoids were extracted as described previously [51] with modifications. Briefly, samples were mixed with polyvinylpyrrolidone and ground to a fine powder in liquid nitrogen. For grape skins, leaves, and tobacco leaves, 5 g of powder was extracted in 25 ml of 0.2 M citrate buffer (pH = 5.0) at 4°C for 16 h in the dark. For grape berries, 5 g of powder was thawed directly at 4°C for 16 h. After centrifugation at 8000 × *g* for 15 min, the clear supernatant was collected for free monoterpenoid analysis.

Glycosylated monoterpenoids were isolated from the supernatant by solid-phase extraction using PepSep columns (150 mg/6 ml; Bonna-Agela Technologies, Beijing, China). The columns were preconditioned with 10 ml methanol, followed by 10 ml water, then loaded with 2 ml of the clear supernatant. After washing with 2 ml water and 5 ml dichloromethane, the glycosylated compounds were eluted with 20 ml methanol. The eluate was then evaporated to dryness at 30°C and the residue was redissolved in 5 ml of 0.2 M citrate–phosphate buffer (pH = 5.0). Enzymatic hydrolysis was performed by adding 100 μl AR2000 (Rapidase, 100 g/l) and incubating at 40°C for 16 h.

For GC–MS analysis, 5 ml of each free or hydrolyzed sample was transferred to a 20-ml vial containing 1.5 g of NaCl and 10 μl of 4-methyl-2-pentanol (internal standard, 1.036 g/l), then sealed with a polytetrafluoroethylene (PTFE)/silicone septum. Terpenoids were extracted by headspace solid-phase microextraction using a divinylbenzene/carboxen/polydimethylsiloxane (DVB/CAR/PDMS) 50/30 μm fiber (Supelco, Bellefonte, PA) under agitation at 40°C for 30 min. Analyses were performed on an Agilent 6890/5975C GC–MS system (Agilent Technologies, Santa Clara, CA, USA) equipped with a 19 091 N-136 HP-INNOWAX column (60 m × 0.25 mm × 0.25 μm, J&W Scientific, Folsom, CA, USA) [31]. Terpenoids were identified based on retention times and quantified using standard calibration curves as described previously [51].

### RNA-seq

Total RNA was extracted from berry samples of two grape cultivars at seven developmental stages using the Polysaccharide/Polyphenol Plant Total RNA Mini Kit (GeneBetter, Beijing, China) according to the manufacturer’s protocol. Forty-two cDNA libraries were constructed and sequenced on the Illumina platform following quality control. After adapter trimming and quality filtering, clean reads were aligned to the grape reference genome PN40024 12X.v3 (https://urgi.versailles.inra.fr/Species/Vitis/Annotations). Gene expression levels were quantified as FPKM. DEGs were identified using the thresholds of |log_2_FoldChange| ≥ 1 and FDR < 0.01. Gene Ontology (GO) and Kyoto Encyclopedia of Genes and Genomes (KEGG) enrichment analyses of UGT gene clusters were performed using TBtools.

### Weighted gene coexpression network analysis

Coexpression network analysis was performed using the WGCNA package in R [52]. Modules were constructed using the automatic ‘blockwise’ function with default parameters except: soft power threshold = 13, minModuleSize = 80, and cutHeight = 0.25. Associations between module eigengenes and monoterpene content were evaluated by Pearson correlation analysis.

To identify genes involved in glycosylated monoterpene biosynthesis, DEGs between ‘Ruiduxiangyu’ and ‘Ruiduhongyu’ at five developmental stages (E-L35–E-L39) were integrated with metabolite data for differentially accumulated glycosylated terpenoids. Correlations between candidate genes and glycosylated compounds were visualized using the ggcorrplot package in R.

### Gene isolation, phylogenetic tree construction, and multiple sequence alignment

The coding sequences (CDS) of candidate VviGTs and VviMYB174 were amplified from cDNA libraries of RDHY and RDXY using gene-specific primers (Supplementary Table S14). Protein sequences of UGTs and R2R3-MYB TFs from *Arabidopsis thaliana* and other species (Supplementary Tables S6 and S13) were retrieved from TAIR (https://www.arabidopsis.org/) and NCBI (https://www.ncbi.nlm.nih.gov/). Phylogenetic trees were constructed using the neighbor-joining method in CLUSTALW (https://www.genome.jp/tools-bin/clustalw) and visualized with Evolview (https://www.evolgenius.info/evolview/). Multiple sequence alignments were performed using DNAMAN 8 (LynnonBiosoft, USA).

### Transient transformation

Recombinant pCAMBIA1301 plasmids were transformed into *Agrobacterium tumefaciens* strain GV3101 for transient overexpression*.*

*Leaves*: the abaxial surface of ‘Marselan’ and ‘Ruidukemei’ leaves was gently punctured with a sterile needle. Leaves were placed abaxial side-up in an *A. tumefaciens* suspension and vacuum-infiltrated for 5 min. *V. quinquangularis* L. leaves were fully immersed in *A. tumefaciens* suspension and vacuum-infiltrated for 30 min at −0.8 MPa. Tobacco (*N. benthamiana*) leaves were infiltrated by syringe injection. After infiltration, *V. quinquangularis* leaves were incubated in darkness for 3 days; tobacco leaves were incubated in darkness for 12 h, then transferred to a 16-h light/8-h dark photoperiod. Both were subsequently treated with 100 μM linalool, nerol, and citronellol, and 10 μM geraniol (in 0.5 M MES/1 M MgCl_2_) for 4 h before harvest.

*Grape berries*: whole berries were infiltrated using the same vacuum-infiltration procedure. For skin-specific expression, berries were washed, pierced 20 times with a sterile needle, immersed in *A. tumefaciens* suspension, and vacuum-infiltrated for 30 min at −0.8 MPa [13]. Treated berries were blotted dry and placed in Petri dishes lined with moist gauze. Skins were collected after 3 days of incubation under a 16-h light/8-h dark photoperiod at 25 ± 1°C.

### Virus-induced gene silencing

Recombined pTRV1 and pTRV2 vectors were transformed into *A. tumefaciens* strain GV3101. Equal volumes of cultures containing pTRV1 and either pTRV2 (empty vector control) or pTRV2-VviMYB174 (silencing construct) were mixed and infiltrated into grape leaves and berries using the transient transformation protocol described above.

### RNA extraction, cDNA synthesis, and Reverse Transcription-Quantitative Polymerase Chain Reactionn (RT-qPCR) analysis

Total RNA was extracted from grape berries, grape skins, grape leaves, and tobacco leaves using the FastPure Universal Plant Total RNA Isolation Kit (Vazyme, Nanjing, China) according to the manufacturer’s protocol. RNA concentration and integrity were assessed using a microspectrophotometer and agarose gel electrophoresis. First-strand cDNA was synthesized from 1 μg total RNA using HiScript II Q RT SuperMix for qPCR (R223, Vazyme, Nanjing, China). RT-qPCR was performed with the ChamQ Universal SYBR qPCR Master Mix (Q711, Vazyme, Nanjing, China) on a CFX96/384 Touch Real-Time PCR Detection System (Bio-Rad, CA, USA). Each sample was analyzed in triplicate. *VviUBIQUITIN* served as the reference gene. Relative expression levels were calculated using the 2^−ΔΔCT^ method. All primers are listed in Supplementary Table S14.

### Heterologous protein expression and purification

The full-length CDS of *VviGTs* were cloned into the pMAL-c5x expression vector with N-terminal MBP-tag using the primer listed in Supplementary Table S14. Recombinant protein was expressed in *Escherichia coli*. Rosetta (DE3) (TransGen, Beijing, China). After growing the precultures overnight at 37°C and 180 rpm in LB medium containing 100 μg/ml ampicillin and 34 μg/ml chloramphenicol to achieve an OD_600_ of 0.6, isopropyl-β-D-thiogalactopyranoside was added at a final concentration of 0.3 mmol/l to induce the protein expression. Cultures were incubated at 16°C and 160 rpm for 16–20 h. Cells were harvested by centrifugation (4000 × *g*, 4°C, 20 min) and were disrupted by sonication. Recombinant MBP fusion proteins were purified using amylose resin (NEB, Ipswich, MA, USA) following the manufacturer’s instructions. Briefly, the crude protein extract was incubated with the amylose resin for 2–3 h to bind MBP fusion protein. Then the nonspecifically bound protein was removed by washing the resin 2–3 times with column buffer. The recombinant proteins were eluted with elution buffer containing maltose and quantified by Bradford reagent (Sangon Biotech, Shanghai, China). The purified protein was confirmed by sodium dodecyl sulfate-polyacrylamide gel electrophoresis (SDS-PAGE).

### UDP-Glo™ Glycosyltransferase Assay

UDP-Glo™ Glycosyltransferase Assay was used to examine the *in vitro* enzyme activity of VviGTs according to the manufacturer’s instructions. The enzymatic reactions were performed in a 200 μl mixture containing 100 mM Tris–HCl buffer (pH = 7.4, with 10 mM DTT), 1 mM UDP-glucose, 500 μM linalool dissolved in dimethyl sulphoxide, and 40–60 μg purified protein. The reaction mixture was incubated at 30°C and 250 rpm overnight. Subsequently, 25 μl of the reaction mixture was transferred to a white 96-well plate, mixed with 25 μl of UDP detection reagent, shaken for 30 s and incubated at room temperature for 60 min. Luminescence was measured with Victor Nivo Multi-Function Plate Reader (PerkinElmer, Waltham, MA, USA). The UDP concentration was calculated based on a standard curve generated from the same assay.

### Yeast one-hybrid assay

Y1H assays were performed using the Matchmaker Gold Yeast One-Hybrid Library Screening System (Clontech, Mountain View, CA, USA). Genomic DNA was isolated from RDHY and RDXY leaves using the Plant Genome Extraction Kit (DP3112, Beijing Bioteke, China). Promoter fragments were PCR-amplified and cloned into the pAbAi vectors to generate bait constructs. The CDS of *VviMYB174* was cloned into pGADT7 vector as prey. Bait vectors were linearized and transformed into Y1HGold to create bait strains. The minimum Aureobasidin A (AbA) concentration required to suppress self-activation was determined for each strain. Prey plasmid (pGADT7-*VviMYB174*) or empty vector (pGADT7) was then transformed into bait strains, and cells were plated on synthetic dropout medium lacking leucine (SD/-Leu) with or without AbA. Growth on medium containing AbA indicated promoter activation by VviMYB174. The primers used for cloning are listed in Supplementary Table S14.

### Statistical analysis

Graphs were generated using GraphPad Prism 8.0 (GraphPad Software, San Diego, CA, USA). Heatmaps and correlation bubble plots were created using the ‘pheatmap’ and ‘ggcorrplot’ packages in R Studio 4.1.3 (http://CRAN.R-project.org/), respectively. Data are presented as means ± standard deviation (SD) for three biological replicates. Statistical significance was determined by two-tailed Student’s *t*-test (^*^P < 0.05; ^**^P < 0.01).

### Accession numbers

Sequences from this article can be queried from NCBI data libraries (NCBI, https://www.ncbi.nlm.nih.gov/) under the following accession numbers: VviGT14 (XM_002285734.4), VviGT7 (XM_019225887.1), VviGT22 (XM_002283003.4), VviGT37 (XM_002273320.3), VviGT74 (XM_010652145.2), and VviMYB174 (XM_002284979.4).

## Supplementary Material

Web_Material_uhag188

## Data Availability

All study data are available within the paper and its supplementary data published online. Raw RNA-seq reads generated in this study have been deposited in the National Genomics Data Centre (https://ngdc.cncb.ac.cn/) under project no. PRJCA053735.
