# Supplementary material for: Integrated omics identifies VviGTs and VviMYB174 as drivers of glycosylated monoterpenoid accumulation in ‘Ruiduhongyu’ grapes
Source: Hortic Res. 2026 May 8;13(10):uhag188. doi: 10.1093/hr/uhag188 (PMC13616493; doi:10.1093/hr/uhag188)
Supplement: Supplementary file 1 [file web_material_uhag188.zip › Supplementary Figure.docx]

**Supplementary Figure**

Supplementary Figure S1. Principal component analysis of monoterpenoids in berries of ‘Ruiduhongyu’ and ‘Ruiduxiangyu’ from different development stages.

Supplementary Figure S2. Venn diagram of differentially expressed genes identified in ‘Ruiduhongyu’ and ‘Ruiduxiangyu’ at E-L35, E-L36, E-L37, E-L38 and E-L39.

**PSPG-box**

Supplementary Figure S3. Protein sequence comparison of ten candidate UGT genes.

Supplementary Figure S4. Gene expression analysis of candidate UGT genes with FPKM ＜ 5.

**C**

**B**

**A**

**D**

Supplementary Figure S5. The sequence comparison and the effects of *VviGT14* transient expression on monoterpenoids. Protein sequence analysis of *VviGT14* in two varieties (**A**). Expression levels of *VviGT14* in OE ‘Ruiduhongyu’ berry (**B**). The contents of free-form (**C**) and glycosylated (**D**) monoterpenoids in transgenic berries. Error bars indicate means ± standard deviations (SD) from three replicates. Asterisk indicates statistical significance by independent sample t-test, *P < 0.05, **P < 0.01.

**A**

**DL 2000**

**CK**

**OEGT22**

**B**

Supplementary Figure S6. The effects of *VviGT22* transient expression in *Nicotiana benthamiana* leaves incubated with a mixture of free monoterpenoids. Expression of *VviGT22* in tobacco leaves (**A**). Glycosylated monoterpenoids accumulation in transgenic leaves (**B**). Error bars indicate means ± standard deviations (SD) from three replicates. Asterisk indicates statistical significance by independent sample t-test, *P < 0.05, **P < 0.01.

Supplementary Figure S7. The effects of *VviGT37* transient expression in ‘Marselan’ leaves on free-form monoterpenoids. Error bars indicate means ± standard deviations (SD) from three replicates. Asterisk indicates statistical significance by independent sample t-test, *P < 0.05, **P < 0.01.

**A**

**DL 2000**

**CK**

**OEGT74**

**B**

Supplementary Figure S8. The effects of *VviGT74* transient expression in *Nicotiana benthamiana* leaves incubated with a mixture of free monoterpenoids. Expression of *VviGT74* in tobacco leaves (**A**). Glycosylated monoterpenoids accumulation in transgenic leaves (**B**). Error bars indicate means ± standard deviations (SD) from three replicates. Asterisk indicates statistical significance by independent sample t-test, *P < 0.05, **P < 0.01

Supplementary Figure S9. UDP-Glo™ Glycosyltransferase Assay of candidate genes. SDS-PAGE analysis of the recombinant proteins VviGT22 (**A**), VviGT37 (**C**), and VviGT74 (**E**). Lanes 1–4 represent total protein after induction, total protein after cell lysis, supernatant protein after cell lysis, and purified protein, respectively. UDP concentration in reaction products of no enzyme (CK), MBP protein or VviGTs protein, along with UDP-glucose and monoterpenoids (**B, D, F**). The standard curve used in the assay (**G**). Asterisk indicates statistical significance by independent sample t-test, *P < 0.05, **P < 0.01.

**A**

**B**

**C**

**D**

Supplementary Figure S10. The effects of *VviMYB174* transient expression on free-form monoterpenoids accumulation in OE ‘Ruidukemei’ leaves (**A**), OE ‘Marselan’ leaves (**B**), OE ‘Sabel’ skins (**C**) and VIGS ‘Marselan’ leaves (**D**). Error bars indicate means ± standard deviations (SD) from three replicates. Asterisk indicates statistical significance by independent sample t-test, *P < 0.05, **P < 0.01.

**A**

**B**

Supplementary Figure S11. Promoter sequence of *VviGT14* cloned from RDHY and RDXY genome (**A**). *Cis*-acting elements of *VviGT14*promoter predicted by PlantCARE (**B**).

**A**

**B**

Supplementary Figure S12. Promoter sequence of *VviGT22* cloned from RDHY and RDXY genome (**A**). *Cis*-acting elements of *VviGT22* promoter predicted by PlantCARE (**B**).

**A**

**B**

Supplementary Figure S13. Promoter sequence of *VviGT37* cloned from RDHY and RDXY genome (**A**). *Cis*-acting elements of *VviGT37* promoter predicted by PlantCARE (**B**).

**A**

**B**

**AT-rich element**

**TCA -element**

**Box 4**

Supplementary Figure S14. Promoter sequence of *VviGT74* cloned from RDHY and RDXY genome (**A**). *Cis*-acting elements of *VviGT74* promoter predicted by PlantCARE (**B**).

Supplementary Figure S15. VviMYB174 do not bind to the promoter of *VviGT14* by yeast one hybrid.

Supplementary Figure S16. VviMYB174 do not bind to the promoter of RDXY-*VviGT74* by yeast one hybrid.
